# CortiWatch: watch-based cortisol tracker

**DOI:** 10.2144/fsoa-2019-0061

**Published:** 2019-09-26

**Authors:** Paul Rice, Sayali Upasham, Badrinath Jagannath, Roshan Manuel, Madhavi Pali, Shalini Prasad

**Affiliations:** 1 Deparatment of Bioengineering, University of Texas at Dallas, Richardson, TX 75080, USA

**Keywords:** chronoamperometry, circadian cycle, cortisol, portable watch

## Abstract

Sweat-based analytics have recently caught the attention of researchers and medical professionals alike because they do not require professionally trained personnel or invasive collection techniques to obtain a sample. The following presents a small form-factor biosensor for reporting physiological ranges of cortisol present in ambient sweat (8–151 ng/ml). This device obtains cortisol measurements through low volumes of unstimulated sweat from the user's wrist. We designed a potentiostatic circuit on a printed circuit board to perform electrochemical testing techniques. The detection modality developed for quantifying sensor response to varying cortisol concentrations is a current based electrochemical technique, chronoamperometry (CA). From the results, the sensor can detect cortisol in the physiologically relevant ranges of cortisol; thus, the sensor is a noninvasive, label free, cost-effective solution for tracking cortisol levels for circadian diagnostics.

## Lay abstract

Cortisol, typically referred to as the ‘stress hormone’, plays a large role in typical body function. Cortisol matches the human sleep cycle, provides alertness, and is excreted by the adrenal cortex. The adrenal cortex is responsible for some of the body's hormonal secretions and can malfunction or fail with age or due to an autoimmune disorder. Our group has developed a wrist worn biosensor to monitor cortisol noninvasively with low volumes of sweat. The required sample is of low volume that no exercise or physical exertion is required for collection. The device allows researchers and users alike to determine cortisol concentration and thus monitor adrenal gland functionality.

Glucocorticoids are often used as a link to establish the proper functioning of the psychoneuroendocrinological pathway in the human body. Cortisol is a steroidal product of the Hypothalamus–Pituitary–Adrenal (HPA) axis. The levels of cortisol in the body are considered crucial to monitor the effect of chronic stress on physiological well-being [1]. Cortisol follows the circadian rhythm in the body, also referred to as the natural sleep–wake cycle. This is associated with the temporal fluctuations of endocrine hormones in the body. Cortisol awakening response (CAR) is a diagnostically relevant phenomenon for tracking cortisol. This response is associated with the rise in cortisol levels postawakening which is preceded by a drop concentration at 3:00 AM when the subject is sleeping. Using CAR response as a reliable assessment tool for determination of adrenocortical activity was first reported by Pruessner *et al*. in the year 1997 [2]. Following that, tracking the CAR response has been considered to be the gold standard for tracking cortisol levels; however, there were challenges associated with tracking the CAR response with the currently available diagnostic systems. Serum and salivary estimates of cortisol are routinely used by healthcare practitioners to quantify concentrations in the body. Venipuncture stress and active sampling of saliva can result in false cortisol spikes because of sampling associated distress [3]. These sampling-related discrepancies caused the International Society of Psychoneruroendocrinology (ISPNE) to convene a panel to discuss the factors affecting assessment of CAR response. A summary of this discussion was reported by Stalder *et al*, which highlights a few of the key points affecting measurements. Sampling times, duration, sample storage and analysis were all listed as potential issues [4]. Sampling times and duration of sampling are crucial to track the diurnal rise and fall of cortisol [5].

Sweat-based cortisol measurement is an attractive alternative for self-monitoring and tracking diurnal patterns of cortisol. Cortisol is expressed in sweat in the range of 8–141 ng/ml. Development of wearable technology has burgeoned to be a promising alternative to conventional laboratory based diagnostic platforms. They have evolved to study and characterize the composition of human eccrine sweat. These wearables are usually a combination of various material substrates, detection probes and transduction mechanisms. The devices have the ability to quantify the present biomarkers for diagnostics [6]. Electrochemical technique is the commonly used detection modality used by the sensors for detection of select biomarkers in target biofluid. Table 1 summarizes the different electrochemical sensors used for biosensing of cortisol. Developing wearable systems for diagnostics especially for endocrinal abnormalities have specific associated challenges, such as obtaining high specificity from ultralow sample volumes, providing rapid analysis for time-sensitive measurement, creating a suitable form-factor for ease of use, and most importantly conducting real time measurements of the sample [7].

**Table 1.** Summary of electrochemical cortisol biosensing technologies.

Sensor description	Range of detection	Detection technique	Ref.
Electroreduced graphene oxide (e-GRO) based immunosensor	0.1 to 200 ng/ml, PBS buffer	Redox probe-mediated chronoamperometry	[8]
SAM-modified IDEs	10pM to 500 nM, PBS buffer	Redox probe-mediated chronoamperometry	[9]
Sonochemically synthesized ZnO-nanostructure based microfluidic sensors	1 pM to 100 nM, saliva	Faradaic EIS	[10]
Aptamer based microfluidic device	30 pg/ml to 10 μg/ml serum, citrate–phosphate buffer	Faradaic EIS	[11]
Affinity based Au electrode immunosensor	1 pg/ml to 150 ng/ml	Chronoamperometry and nonfaradaic EIS	This work

e-GRO: Electroreduce graphene oxide; EIS: Electrochemical impedance spectroscopy; IDE: Interdigitated electrodes; PBS: Phosphate-buffered saline.

CortiWatch offers a unique platform for tracking cortisol concentrations using human eccrine sweat and a watch-style form factor. The watch performs sensitive detection of cortisol within the physiologically relevant ranges. The sensor uses low volumes of sweat, through passive eccrine sweat and the device is designed to prevent evaporation during sample collection. The purpose of the CortiWatch platform is to perform a 9 h reading to capture rise and fall in the cortisol levels and to be disposable but multiread sensor. As described in the work below, this novel portable device can be used to develop a circadian profile for the user, facilitate self-monitoring and, help improving lifestyle by keeping an eye on the cortisol levels present in the body.

## Materials & methods

### Materials & reagents

Polyamide substrates were obtained from GE Healthcare Lifesciences (NJ, USA). Linker and solvents, namely DSP (Dithiobis [Succinimidyl Propionate]), DMSO (Dimethyl Sulfoxide) and PBS (phosphate-buffered saline), were purchased from Thermofisher Scientific, Inc. (MA, USA). Cortisol (Hydrocortisone) and capture probe (α-cortisol antibody) were obtained from Abcam (MA, USA). Synthetic sweat was prepared in Mili-Q pore deionized water (18 MΩ) according to the protocol described in Mathew *et al*. [12]. Human subject participation was conducted in accordance with the protocol approved by IRB at the University of Texas at Dallas.

### Fabrication for CortiWatch platform

Gold was deposited onto polyamide substrates using the Cryo E-beam evaporation (give tool name). The surface of the electrode was treated using thiol-gold affinity. A total of 5 μl of 10 mM DSP solution was drop casted on gold surface and incubated for 90 min. A total of 10 μg/ml of α-cortisol antibody was then dispensed on the electrode surface and incubated for 15 min. A wash step with PBS was performed to remove physiosorbed molecules. The baseline was recorded with blank PBS and the sensor was then tested using varying cortisol doses (ng/ml) via chronoamperometry (Gamry Reference 600 Potentiostat) to generate calibrated dose response curves for characterizing the platform's sensitivity. Nonspecific signal was captured to quantify the noise of the system caused by interferent molecules. This is illustrated in Supplementary Figure 1. The signal is lower than signal-noise threshold of the system indicating the system is specific for cortisol. For the continuous study, the subject wore the functionalized device for a period of 9 h and hourly measurements were recorded.

### Fourier transform infrared spectroscopy-attenuated total reflectance experimental setup

Fourier transform infrared spectroscopy (FTIR) was the characterization tool used to validate the functionalized immunochemistry on the electrode. The Thermo Scientific Nicolet iS50 FTIR was used in attenuated total reflectance mode. The tool has a germanium attenuated total reflectance crystal, a deuterated triglycine sulfate (DTGS) detector and a KBr window. The sample was prepared on a gold deposited glass substrate with washing steps. A total of 256 scans were performed with a contact area of 1 cm^2^ and a spectrum wavelength range of 675–4000 cm^-1^.

### Portable cortisol measuring system

**Figure 1.**
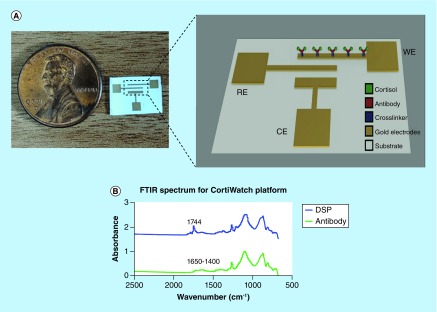
CortiWatch electrode design and functionalization chemistry. **(A)** Picture of the CortiWatch sensor displayed next to a penny for size reference. Electrochemistry schematic of the electrode functionalization. **(B)** FTIR spectrum displaying absorbance peaks related to linker and capture probe (antibody) group for CortiWatch platform. CE: Counter electrode; FTIR: Fourier transform infrared spectroscopy; RE: Reference electrode; WE: Working electrode.

Chronoamperometry was used with the potentiostatic circuit to create a two and three electrode capable system for electrochemical testing and analysis. The system was designed to be a robust and modular rigid printed circuit board (PCB). The three-electrode system schematic is described in Figure 1A. The interdigitated electrode design was chosen as the sensing platform because it offers optimal performance for sweat-based biosensing. The functionalized sensor was then paired with the PCB, which was programed to carry out chronoamperometric measurement equivalent to cortisol concentration from eccrine sweat. Benchtop measurements were performed using Gamry Reference 600 Potentiostat (Gamry Instruments, PA, USA). Our system was developed as a ‘portable potentiostat’ to enhance accessibility as a wearable form-factor device. Further details on the device, along with the block diagram, are presented in the results section.

### Calibrated-dose response study using chronoamperometry

Chronoamperometry was used as the detection modality to characterize the cortisol binding response. Protocol for functionalization of electrode was followed as described in fabrication. A step voltage of 150 mv was applied and measurements were recorded for the duration of 60 s. The current peaks in response to the step potential applied were extracted to characterize dose-dependent response of the CortiWatch sensor.

### Statistical analysis

Data are represented as average ± standard error of the mean. Box plots for on-body data were calculated with 95% CI and are represented as box-whisker plots. Statistical analysis was carried out using Graphpad Prism 7.03. (Graph Pad Software, Inc., CA, USA)

### Human subject studies

Human subject studies were performed according to the protocol no. IRB 18–116, approved by the IRB board at UT Dallas. A patch on the wrist was cleaned with an alcohol swab and the CortiWatch was placed on the cleaned surface of the wrist of the participant. Sensors were prepared with the crosslinker and antibody before being mounted on the subject. A PBS wash was also performed to collect a baseline and ensure that the sensor was working properly. The sensor was then worn and was saturated by passive eccrine sweat from the subject. The device uses a cartridge-style system to interface with the skin and to isolate the sensor from the outside air. The device was not removed from the subject during collection; rather, it was plugged in to a computer and data were collected. The CortiWatch collects and analyzes data.

## Results & discussion

### CortiWatch sensor design & immunochemistry

The CortiWatch sensor platform is designed to be of a wearable form-factor for self-monitoring cortisol levels. The interdigitated electrode design is chosen for the watch-based platform because of its optimum wearable performance. A typical sensing platform has the following components: biorecognition element, transducer, signal converter and amplifier [13]. The developed sensor is an affinity based electrochemical sensing platform. A schematic of the functionalized immunochemistry is depicted in Figure 1A. The biorecognition element is a cortisol antibody. A thiol linker is used to anchor the antibody to the surface of the gold electrode via chemical affinity of sulfur to gold. The capture probe is then immobilized and used for detecting cortisol.

Functionalization of cortisol was confirmed using FTIR. FTIR maps the elements on the functionalized surface using its chemical bond signature as a function of absorbance [14]. The absorbance spectrum for the linker molecule and recognition element is illustrated in Figure 1B. The peaks relevant to functionalization elements are summarized in Table 2. The peak at 1744 cm^-1^ indicates the presence of the NHS ester group for DSP. The introduction of antibody on the DSP functionalized surface leads to a chemical reaction where the NHS ester bond breaks to form amide bonds with the antibody. These amide bonds can be seen as smaller multiple peaks between 1650 and 1400 cm^-1^ in the antibody spectrum depicted in Figure 1B (bottom). The presence of amide bonds confirm the cortisol antibody functionalization on the electrode surface [11,12].

### CortiWatch platform & electronics interface design

**Figure 2.**
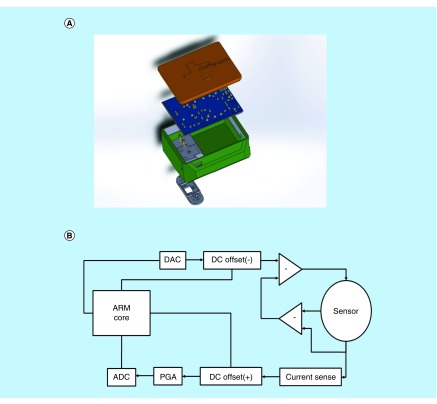
CortiWatch Device render and block diagram. **(A)** Device rendering displaying different components inside the CortiWatch. **(B)** Block diagram of the circuit used in CortiWatch. ADC: Analog to digital converter; ARM: Advanced RISC Machine; DAC: Digital to analog converter; DC: Direct current; PGA: Programmable gain amplifier; RISC: Reduced instruction set computing.

A rendering of the cortisol sensing device is illustrated in Figure 2A. The system is placed in a 3D printed case, where the bottom layer enables point of contact with the epidermal layer for eccrine sweat sample interaction. The sensor is placed in a cartridge-style system under the main case and interfaces with the PCB using gold plated pogo pins. Low volumes of eccrine sweat are needed for sensing. This is amenable to nonactive sweating rates with a total volume of 3–5 nl/gland/min rate according to the sweat gland distribution on the body. This typically yields 3–5 μl of sweat [15,16]. In turn, the device uses a low volume of 5 μl for biosensing; therefore, the user does not have make any active effort for sample collection. The printed circuit board is placed inside the housing and is soldered to the pogo pins for optimal connectivity. A block diagram for the system is represented in Figure 2B.

The two-layer PCB was designed to meet the sensor's measurement needs. This device is similar to the work published by our group in Kinnamon *et al*., in the sense that it mobilizes a potentiostatic biosensing platform [7]. This device was also used for cortisol detection but involves a different electrode configuration and material. The electrochemical testing method has also changed in the presented device. The presented design decreases the overall device size and improves the output signal resolution. In the CortiWatch, an M4-Cortex based ARM processor was utilized for its high clock speed and vast number of peripherals. The microcontroller's onboard 12-bit digital to analog converters (DACs) and analog to digital converters (ADCs) were used for signal generation and collection, respectively. The stimulus for the chronoamperometry test was a 150-mV square wave. The system is run by multiple operational amplifier-based circuits and is inspired by a novel potentiostat model [17]. The AD8608, a four channel operational amplifier, was used to create a set of differential op-amps and was paired with another onboard 12-bit DAC to generate a negative direct current (DC) offset for the square wave signal, fixing it just above zero. The remaining channels of this operational amplifier (modeled in the block diagram) were used to create a sensing modality that mimics the benchtop potentiostatic system. The paired differential amplifiers isolate the current response at the working electrode. This, in turn, allows us to analyze the reaction specific current response. The current was then measured with a shunt resistor. The input and output signals were then positively offset using another AD8608 and were fed through their own LTC6910 programable gain amplifiers. The programable gain amplifiers are digitally stimulated by the microcontroller in response to peak current changes measured in software prior to data collection. This allows the device to use as much of the voltage range as possible, improving the output signal resolution. These amplified signals went to two onboard ADCs for data recording.

### Validation & testing for cortisol response using sweat analog

#### Benchtop assay for cortisol using sweat analog

**Figure 3.**
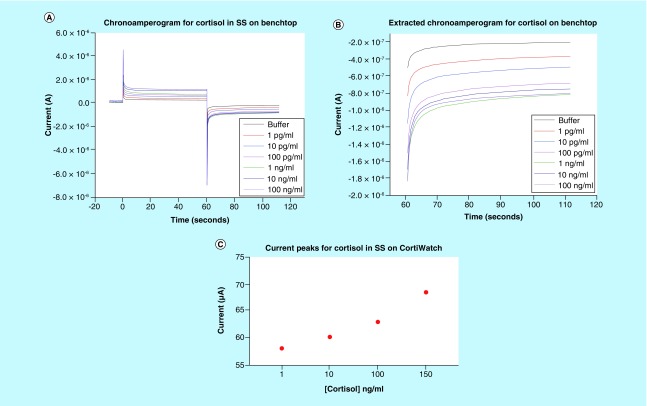
Benchtop sensing response for cortisol. **(A)** Chronoamperogram for cortisol doses ranging from 1 pg/ml to 100 ng/ml in synthetic sweat pH 6 performed on benchtop. **(B)** Extracted negative chronoamperogram for benchtop cortisol studies displaying dose dependent changes. **(C)** Extracted chronoamperometry current peaks for cortisol doses in synthetic sweat for test performed on CortiWatch. SS: Synthetic sweat.

The functionalized sensor system was tested to quantify the cortisol concentration dependent sensitivity using chronoamperometry. As discussed earlier, chronoamperometry is an electrochemical technique that perturbs the system using a step voltage input, resulting in a current peak at the output. This peak is followed by an exponential decay in current [18]. Physiologically, the pH of human sweat is approximately pH 6 [15]. A sweat analog with pH 6 was created to optimize the sensor dose response. All preparation follows the protocol described in the methods section. Synthetic sweat helps map the cortisol response in an environment that simulates human sweat interaction without the presence of interferent molecules. Chronoamperogram for the dose response study is depicted in Figure 3A. The sensor platform was tested for cortisol concentrations ranging from 1 pg/ml to 100 ng/ml, which is within the physiological range [17]. We can observe from the graph that there is a dose dependent response. The steady state current decreases as cortisol concentration increases. The current peak sensitivity can be attributed to the binding response between cortisol and the functionalized capture probe. The binding leads to redistribution or rearrangement of charges in the electrical double layer, which is formed when a polarized electrode comes in contact with an electrolyte [16]. Since there is no external redox probe involved, the rearrangement is reflected as capacitive modulations in this double layer. These capacitive modulations are then extracted as a chronoamperogram.

A simplified chronoamperogram for the cortisol sensor with a negative bias for the cycle is depicted in Figure 3B. From this graph, a clear dose-dependent response coupled with significant current changes can be observed. The buffer represents the baseline reading where only blank buffer with no cortisol is added. Formation of cortisol antibody–cortisol complex dictates the curve generation for the sensor system [19,20].

#### CortiWatch assay for cortisol using sweat analog

The sensor response for cortisol was evaluated using the portable CortiWatch platform. Cortisol concentrations ranging from 1 to 150 ng/ml were spiked in human sweat analog to simulate the physiologically relevant range. The chronoamperogram is depicted in the Supplementary Figure 2. The current peaks for the output were extracted and plotted as a function of concentration. This is illustrated in Figure 3C. It can be observed that the peak current value ranges between 58 and 70 μA. This change agrees with the previous benchtop results that we obtained from Gamry Potentiostat system. Similarly, we can observe that with the current change, a wider dynamic range for cortisol is present for CortiWatch.

### Validation of sensor's cortisol sensitivity using alternate techniques

#### Validation using ELISA

**Figure 4.**
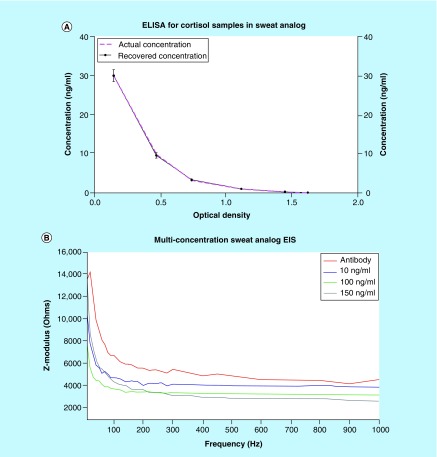
Sensor response validation. Validation of sensor's cortisol response using **(A)** ELISA and **(B)** Electrochemical Impedance spectroscopy for varying cortisol dose concentrations. EIS: Electrochemical impedance spectroscopy.

ELISA is a commercially available technique used by physicians for diagnosing cortisol level-related abnormalities. ELISA is an optical immunoassay-based technique where concentration are quantified as a function of absorbance of the system [21]. A commercially available cortisol kit from Salimetrics (PA, USA) was procured and artificially spiked with cortisol samples to evaluate their recovery. This result is illustrated in Figure 4A, where the pink-dotted line represents actual concentration and solid-black line represents the recovered concentration. This indicates the spiked samples are recovered with a correlation factor of 0.99 and there is no loss in concentration during the sensor testing period; thus, the sensor response for cortisol can be attributed to actual binding between cortisol and the functionalized system present on the electrode.

#### Cortisol response evaluation using electrochemical impedance spectroscopy

An alternate electrochemical technique, electrochemical impedance spectroscopy (EIS) was used to test the cortisol sensitivity of the sensor. Similar to the previous results, the sensor was tested with physiologically relevant concentrations of cortisol spiked in sweat analog. With EIS, we can map the frequency-dependent impedance response to cortisol concentrations. Impedance is a resistive phenomenon which involves quantifying the resistive and capacitive components of a system. This is done by taking the input voltage and dividing it by the output current. At lower frequencies, we can observe the binding response at the interfacial surface of electrode–electrolyte [22]. The results shown in Figure 4B display the impedance response over an input frequency range of 10–1000 Hz. With each increment in cortisol concentration, the impedance of the system decreases as observed by the Zmod changes on the y-axis. Using EIS, we can extract the binding changes in real time during the frequency sweep [23]. These results confirm the finding that the sensor system capable of detecting and responding according to the cortisol concentration present in the system. Because impedance and current are inversely proportional, these results are in accordance with the chronoamperometry data previously shown.

### CortiWatch on-body testing

**Figure 5.**
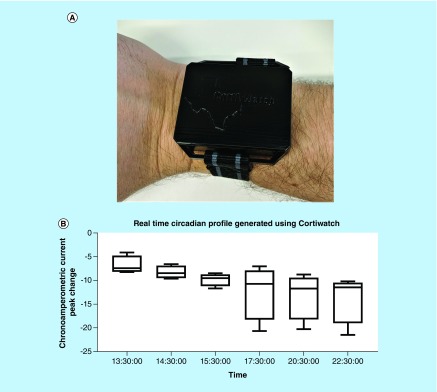
On-body testing using CortiWatch. **(A)** Wearable case design for CortiWatch placed on human subject during trials. **(B)** Real-time circadian profile for human participant generated using CortiWatch for a 9 h time period.

Following the extensive benchtop testing, the sensor was tested on the human body to track the circadian rise and fall of cortisol. Trials were conducted over 4 days on one human subject to display repeatability. Initially, the sensor baseline was determined following the functionalization of the capture probe and prior to mounting the sensor on the subject. The obtained baselined was used as a reference to quantify the cortisol for further measurements throughout each trial. The sensor was then placed on the human subject and hourly measurements were taken for 9 h. The sensor was intactly placed in a case design to ensure steady contact with the skin on the subject and prevent any environmental artifacts such as evaporation of sweat throughout the duration of the test. Variability expressed as error bars is expected while performing human subject data, and can be attributed to humidity present in the air, temperature fluctuations. However, we still see a change for cortisol level decrease in the response of the sensor. According to the circadian cycle and duration of the experiment, the cortisol concentration is constantly decreasing throughout the duration of the study [24]. This is evident from the correlated current change with time in Figure 5. The four trials were plotted with 95% CI. Variability expressed in the plot is expected while performing human subject data, and can be attributed to humidity, temperature fluctuations. However, we still see a trend in the response of the sensor which mirrors the body's cortisol level decrease. Tracking this circadian profile will be useful for noninvasive monitoring of cortisol and can be used to track the diagnostically relevant time points, in this particular case, the sensor demonstrates the rise in cortisol concentrations over a period of 9 h [25]. This novel portable CortiWatch demonstrates proof of feasibility of using a passive epidermis sensing for tracking cortisol concentrations for long-term temporal studies for quantification of stress present in the body at any given time.

## Conclusion

In this work, we have demonstrated the detection of cortisol using a portable watch form-factor sensing platform. This immunoassay-based affinity biosensor performs electrochemical detection of cortisol in the physiologically relevant ranges using chronoamperometry. This detection modality and the sensor system showed sensitivity to varying cortisol concentrations in the form of output current. Alternate techniques like ELISA and EIS were used to confirm the findings and it was established that the sensor response is correlated to cortisol concentration present in the system. On-body testing was performed, and circadian cycle was tracked for a 9 h window which indicates the stability and long-term reliability of the sensor for cortisol monitoring.

## Future perspective

Future perspective of wearable cortisol technologies will incorporate translatability into developing a point-of-need rather than point-of-care monitoring system for adrenal or circadian dysfunction diagnostics. There are still challenges for mapping the entire circadian cycle and using it as a substitute for ELISA; however, this work is the proof that self-monitoring cortisol levels can be possible. Similar innovations in this field would facilitate early diagnosis of lifestyle disorders and make them manageable and promote healthier lifestyle. 

**Table 2.** Fourier transform infrared spectroscopy absorbance peaks corresponding to respective functional groups.

Wavenumber (cm^-1^)	Functional group
1744	NHS ester group for DSP
1372	CH_3_ bending for alkane chain of DSP
1540	Amide-I bond for antibody
1650	Amide-II bond for antibody

**Summary points**

- Sensor platform was characterized for cortisol-specific functionalization using Fourier transform infrared spectroscopy.
- The device houses a portable potentiostat and the components and novelty of the components were discussed.
- Sensor was tested using a sweat analog for sensitivity for cortisol on both, benchtop and using the CortiWatch, which indicated that the sensor was able to detect cortisol.
- Sensor's sensitivity for cortisol was validated using alternate techniques and finally real-time circadian profile for human participant was generated using the Cortiwatch. Thus, CortiWatch can be successfully used for tracing cortisol and facilitate self-monitoring.
